# Magnetic vortex chirality determination via local hysteresis loops measurements with magnetic force microscopy

**DOI:** 10.1038/srep29904

**Published:** 2016-07-18

**Authors:** Marco Coïsson, Gabriele Barrera, Federica Celegato, Alessandra Manzin, Franco Vinai, Paola Tiberto

**Affiliations:** 1 INRIM, strada delle Cacce 91, 10135 Torino (TO), Italy; 2 Università di Torino, via P. Giuria 7, 10125 Torino (TO), Italy

## Abstract

Magnetic vortex chirality in patterned square dots has been investigated by means of a field-dependent magnetic force microscopy technique that allows to measure local hysteresis loops. The chirality affects the two loop branches independently, giving rise to curves that have different shapes and symmetries as a function of the details of the magnetisation reversal process in the square dot, that is studied both experimentally and through micromagnetic simulations. The tip-sample interaction is taken into account numerically, and exploited experimentally, to influence the side of the square where nucleation of the vortex preferably occurs, therefore providing a way to both measure and drive chirality with the present technique.

Magnetic vortices in patterned magnetic materials have attracted a widespread interest in recent years^1^,^2^, especially for their perspective applications in magnetic storage^3^ or as microwave oscillators^4^. Much of the attention has been devoted to the control of the vortex chirality, as a system for permanently storing information. Several techniques have been proposed, by exploiting electrical means such as rotating fields^5^ or field impulses^6^,^7^ that could be implemented in integrated devices, by intervening on the shape of the patterned nanostructures to break their symmetry and induce a preferred chirality^8^,^9^, or by approaching the subject from a more fundamental point of view via the analysis of the mutual interactions between a vortex structure and a magnetic force microscope tip^10^,^11^,^12^. As important as the control of the vortex chirality is the possibility to retrieve the stored information by measuring (i.e. reading) the chirality; to this purpose, experiments exploiting techniques like X-ray magnetic circular dichroism (XMCD)^13^, SEM with polarisation analysis (SEMPA)^9^, planar Hall effect^14^, magneto-resistance^3^,^15^ and magnetic force microscopy (MFM)^10^ have been proposed, as well as nanopatterned devices like lateral spin valves^16^. However, some of these techniques do not provide the evolution of the magnetisation with the applied field, or are rather complex or not generally available in laboratories. MFM is a relatively common technique that has been successfully used to characterise the vortex chirality in magnetic patterned structures displaying either perpendicular^17^ or planar anisotropy, in the latter case even as a function of an applied magnetic field^18^,^19^,^20^.

In this paper, we demonstrate the possibility of determining vortex chirality from “local hysteresis loop” measurements, by means of a recent MFM technique based on a local characterisation of sub-micrometric structures^21^. When measuring hysteresis loops by means of magnetometers (e.g. SQUID) or MOKE, the information on vortex chirality is typically unavailable, as the hysteresis loops normally contain only the information on the projection of the magnetisation along the direction of the applied field, that is independent of the actual chirality state. However, with the technique exploited here, information on vortex chirality is fully available for both loop branches, together with a complete field dependence of the magnetisation behaviour in the square dot.

## Materials and Methods

### Samples fabrication

Square dots of Ni_80_Fe_20_ have been prepared by rf sputtering on SiO_2_ substrates a continuous thin film with a thickness of 30 nm. Subsequently, electron beam lithography has been used to impress the desired geometry on a negative resist: after its development, square dots of resist with a lateral size of 800 nm and arranged in an array remain on top of the metallic film. Finally, sputter etching in Ar removes the unprotected Ni_80_Fe_20_ film. After chemical removal of the remaining resist, the magnetic square dots array remains on the substrate. The array consists of 3 × 3 squares separated by an interdot distance of more than 2 *μ*m to avoid mutual magnetostatic interactions. A SEM image of one row of the array is shown in Fig. S1.

### Local hysteresis loops measurements

In order to measure a local hysteresis loop^21^, a magnetic thin film patterned in sub-micrometric elements is imaged in normal MFM mode. In our case, the microscope is a Bruker Multimode V Nanoscope 8 system, with non magnetic scanner, head and tip-holder. Then, a profile of an individual element of the pattern is chosen, and the slow scan axis of the microscope is turned off. In this way, the same profile is repeatedly acquired over time, in both its height (pass 1, morphology) and phase (pass 2, magnetic signal) channels. The choice of the profile is arbitrary, as discussed in ref. 21, but for the purpose of investigating the effect of magnetic vortex chirality on local hysteresis loops it is necessary that the chosen profile is not a symmetry axis of the patterned structure. For example, in the case of the 800 nm squares that we investigate here, profiles parallel to two of the square dot edges, at approximately 50 nm from the closest border, are adequate; the exact position of the profile is not extremely important, and it is better to chose a profile free from significant morphological imperfections that could induce artefacts in the magnetic image.

Each time the profile is acquired, the magnetic field, applied in-plane and parallel to the scan direction and to two of the square edges, is changed, thanks to a proper synchronisation with the end-of-line signal of the microscope controller^21^. In this way, a single image consisting of *n* (e.g. 512) lines is constituted by *n* repetitions of the same profile, each acquired at a different applied field. By performing a suitable field history, local hysteresis loops of the imaged patterned structure can be obtained. In order to achieve this result, a suitable analysis of the acquired image has to be performed^21^. In our case, we measure the phase difference (“phase contrast”) between the left and right edge of the square in each scanned profile (i.e. for each applied field value), obtaining the local hysteresis loop of the square.

The MFM tip is prepared in a vertical magnetisation configuration (i.e. its magnetisation is parallel to its axis and to its vibration direction, and perpendicular to the sample surface), therefore it is sensitive to the second derivative of the vertical component of the field generated by the sample magnetisation with respect to the vertical direction. As during the local loops measurement the tip is subjected to external fields, it is important to take into account their possible effects. The applied field is parallel to the sample surface and perpendicular to the tip magnetisation axis: significant, irreversible rotation of the tip magnetisation along this direction will happen only when a significant energy barrier is overcome; this is a consequence of the elongated shape of the tip, which introduces shape anisotropy effects that drive the magnetisation to point parallel to the tip axis. It is also important to choose tips with a magnetic coating with a sufficiently high coercivity. Even though the nominal coercive field of the tips commercially available is an indication of the limit above which their magnetisation will flip along the tip axis, tips with higher coercivity will be certainly less affected also by fields parallel to the sample plane, as in our case. We used Co-Cr coated commercial HR-MESP tips with a nominal coercive field of ≈900 Oe. We observed a loss of magnetic contrast using these tips under applied in-plane fields above 1000 Oe, which resulted in a permanent rotation of the magnetisation off the tip axis. In our measurements, the applied field (maximum value 800 Oe) has always been kept well below this limit, and the magnetic contrast has never been lost.

Finally, it is important to briefly discuss the possibility of calibrating the MFM phase signal in order to get a quantitative determination of the fringing field value that is sensed by the tip. This procedure is possible, although complex^22^,^23^,^24^,^25^,^26^, but not really required in our case. The calibration procedure allows to convert the phase signal (measured in degrees) into a 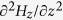 signal (measured in A/m^3^), leaving the colour representation of the MFM image otherwise unchanged. The “phase contrast” determined with the local hysteresis loops measurements will then simply change units, but its applied magnetic field dependence will otherwise remain the same. As we will see, any information that we can extract from the local hysteresis loops measurements concerning vortex chirality in the studied square dots does not require the MFM tip to be calibrated; for this reason, in the following the phase channel in pass 2 and the phase contrast will be expressed in arbitrary units.

### Micromagnetic simulations

Micromagnetic simulations have been performed on a square dot of Ni_80_Fe_20_ having a lateral size of 800 nm and a thickness of 30 nm, using standard parameters for this alloy (*M*_*S*_ = 860 kA/m, exchange constant of 13 pJ/m and negligible magnetocrystalline anisotropy). The spatial configuration of the magnetisation in the square is calculated as a function of the applied field by solving the Landau-Lifshitz-Gilbert (LLG) equation on a mesh of hexahedra with average size comparable to the exchange length; in each mesh element, the magnetisation and the effective field are considered uniform. The used micromagnetic solver is described in details in refs 27, 28, 29, 30.

Micromagnetic simulations start at zero applied field, then the field is increased in the sample plane up to 800 Oe and then it is reduced in steps of 12.566 Oe (1 kA/m) down to −800 Oe. At each field step, the time evolution of the magnetisation vector is calculated by means of a Cayley transform based scheme^29^,^30^ until equilibrium is reached (maximum local value of the torque exerted by the effective field below a given threshold, see ref. 31 for details). From the equilibrium configurations of the magnetisation, the corresponding simulated MFM images are reconstructed by assuming a tip uniformly magnetised along the vertical axis (a magnetic dipole) and not affected by the stray field of the sample or by the applied magnetic field. The agreement between the simulated and experimental MFM images prove these assumptions are correct. Conversely, the effect of the tip magnetisation on the sample magnetic domain configuration may or may not be taken into account, at choice. MFM reconstructed images result from the calculation of the spatial distribution of the second order derivative of the vertical component of the square dot stray field, with respect to the vertical direction, at a distance of 50 nm from the square surface. Finally, each point of the simulated MFM image is averaged with its neighbours up to a distance of 35 nm, which corresponds to the nominal radius of the tip used in the experiment, to take into account the averaging effect resulting from a tip having a finite size.

### Monte Carlo simulations

The process that drives the evolution of the magnetisation from the (quasi) symmetric state that precedes the formation of the C state and the C state itself is complex, and its detailed modelling overcomes the aim of these Monte Carlo simulations. However, as outlined in the Discussion section, the expected equiprobability of these two chirality states is not observed in our experiments. In order to assess whether this effect could be ascribed to the tip-sample interaction, a very simple model has been applied, which consists in imagining that during the infinitesimal variation of the applied magnetic field that triggers the formation of the asymmetric C-state from the symmetric state that precedes it, the physical system is very roughly described by a double well. Each well represents one chirality of the C-state. The barrier between the two wells is initially low, so that the system can randomly fluctuate between the two chirality states. However, during the infinitesimal field variation the two wells become progressively deeper, or the barrier between them progressively taller, so that random jumps from one chirality state to the other become progressively less probable. Eventually, the barrier will be high enough that the random fluctuations will not be able to let the system change its state, and the square will develop a stable C-state, and a vortex, with a well defined chirality, corresponding to the well into which the system has remained at the end of this process. This can be considered the final equilibrium configuration of the magnetisation at the end of this process.

This sequence of events is reproduced by a Monte Carlo simulation, that defines two wells having a certain height *h*_1_ an *h*_2_ with 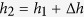, Δ*h* being the asymmetry (possibly zero) between them. An additional parameter *T* defines the upper threshold energy of the random events of any kind that can lead the system flip from one possible chirality configuration to the other one. In the initial state, when the process begins that will lead to the formation of an asymmetric state in the magnetisation of the square, *h*_1_ = 0 and *h*_2 _= Δ*h*, and the system is placed randomly in one of the two wells. For each iteration of the Monte Carlo algorithm, a certain number of random events are generated; each event has an “energy” randomly chosen between 0 and *T*. If this value is larger than the depth of the well in which the system is placed at that time, the system will jump to the other well, otherwise it will remain in the previous state. Then, both *h*_1_ and *h*_2_ are increased by the same amount, and a new iteration is performed, with slightly deeper wells. The loop ends when the wells have become deeper than *T*, so that no event can induce the system to flip its chirality any more: the system is now in a stable chiral state. Its chirality is stored in a counter (*w*_1_ and *w*_2_ are incremented when the system ends being in well 1 and 2 respectively), and the whole process is repeated from the beginning a large number of times. Eventually, 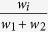 will give the probability that the square has to develop a chirality of type 1 or 2 (e.g. clockwise or anticlockwise) for a given Δ*h* asymmetry between the two wells.

Δ*h*, in this very simple model, represents the tip-sample interaction; if the tip is assumed to be able to induce a preferential chirality state in the square because of its fringing magnetic field, then one of the two wells (*h*_2_ in our case, for positive Δ*h* values) will be deeper than the other, by a certain amount Δ*h*. The whole Monte Carlo simulation can be repeated for Δ*h* values starting from zero and increasing up to 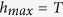 (larger values would be meaningless, as once the system falls into the deeper well, it will no longer jump out of it). In the end, the probability that the system has to develop any of the two chirality states can be calculated as a function of the Δ*h*/*h*_*max*_ ratio, which represents the intensity of the tip-sample interaction with respect to the processes of any kind that can induce a chirality reversal in the square during the formation of the asymmetric C-state from the symmetric state that precedes it.

## Results

**Figure 1:**
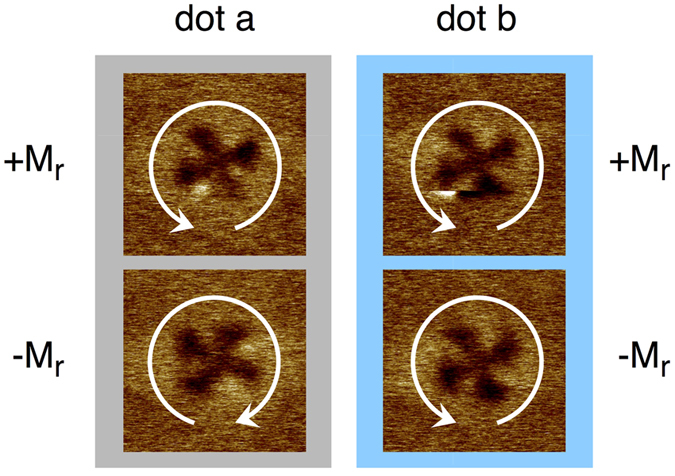
MFM images of two different squares at their positive ( + *M*_*r*_) and negative (−*M*_*r*_) remanent states. In square “a” the chirality is inverted between the two remanent states, in square “b” it is preserved.

A portion of the square dots array has been imaged by MFM after having applied a positive and saturating, or negative and saturating magnetic field. Two representative squares are shown in Fig. 1, displaying the two possible remanent states +*M*_*r*_ and −*M*_*r*_ (respectively, magnetisation at zero applied field coming from positive or negative saturation): a magnetic vortex whose chirality is either inverted (square “a”) or preserved (square “b”) between the positive and negative remanent states. A vortex configuration is to be expected at the remanence in the case of dots having either circular or square shape having diameters of several hundred nanometers^32^,^33^,^34^,^35^,^36^. It is important to point out that the actual chirality states displayed by the square dots of Fig. 1 at their remanence do not remain the same when they are brought to remanence from saturation in subsequent times; even though imperfections in dots shape or on their surface may induce preferred chirality states^37^, in our samples they do not seem to univocally determine the vortex chirality. Identifying the two possible clockwise and anticlockwise remanent states may not be easy in individual MFM images, but it is simpler when two different states are compared. In the case of square “a”, in fact, the bright - dark colours alternate from the top-left corner of the square in dark-bright-dark-bright-etc. sequence in the positive remanence state. Conversely, in the negative remanence state, starting from the same corner of the square, the colours sequence is bright-dark-bright-dark-etc. Even though the clockwise or anticlockwise direction of the chirality represented by the arrows in Fig. 1 may only be conventional (if the actual polarity of the MFM tip is ignored or neglected), the reversal of the colour sequence indicates opposite chirality states for the case of square “a”. In the case of square “b”, instead, it is easy to verify that in the two remanence states the vortex chirality is the same. This behaviour is in principle stochastic, driven by thermal fluctuations, meaning that the vortex chirality can be clockwise or anticlockwise with the same probability each time each square is brought to the remanent state from magnetic saturation, unless events breaking the symmetry take place (asymmetric shapes^38^, nearby magnets^39^, defects^40^).

**Figure 2:**
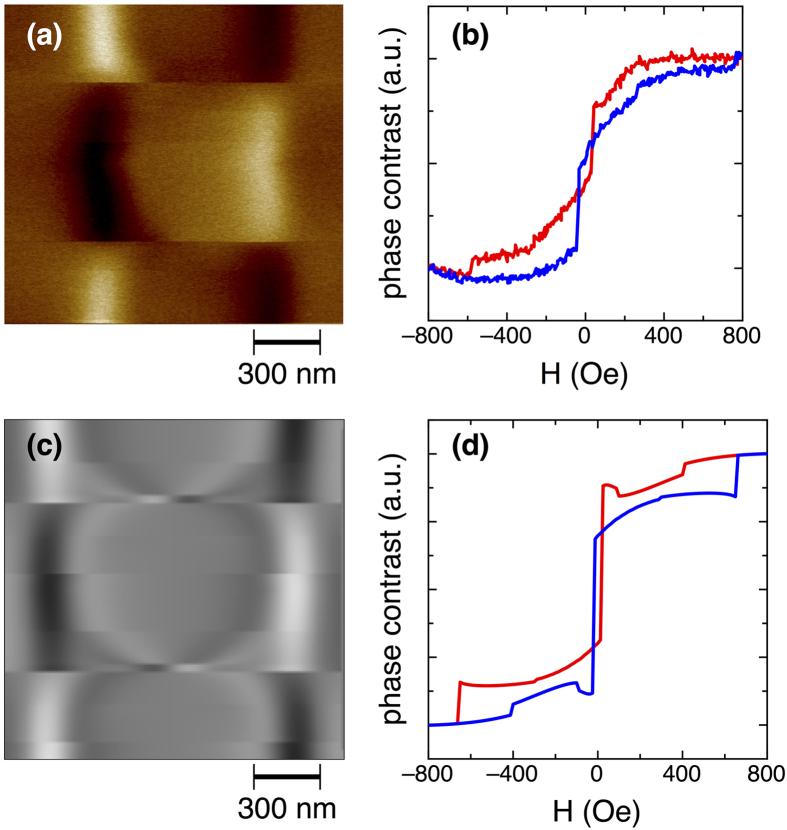
Local hysteresis loops: experimental (top row) and simulated (bottom row) results. (**a**,**c**) MFM images constituted by the same profile acquired multiple times as a function of the applied magnetic field. (**b**,**d**) corresponding local hysteresis loops.

Following the procedure discussed in the “Materials and Methods” section and described in details in ref. 21, local hysteresis loops can be measured on a single magnetic square under the application of an in-plane magnetic field. The scan profile for the measurement of the loop has been chosen approximately at 50 nm from the bottom edge of the square; however, its precise position is not critical. An example of a local hysteresis loop measurement is shown in Fig. 2, where the experimental results are compared with micromagnetic simulations^27^,^28^,^31^, as discussed in ref. 21.

## Discussion

**Figure 3:**
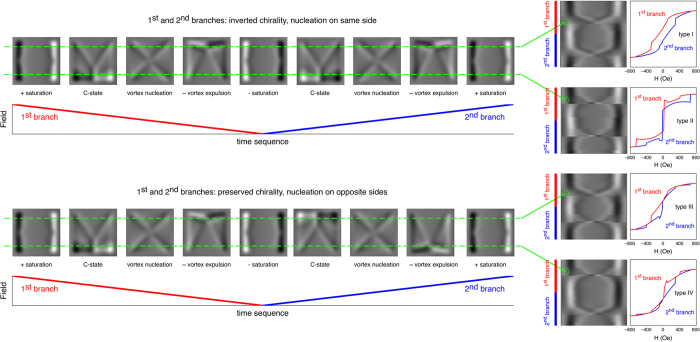
Simulated MFM images and local hysteresis loops of a square dot. Dashed green lines represent the line scanned by the MFM tip. Top row: symmetric loops corresponding to inverted chirality (the vortex nucleates on the same side in both loop branches). Bottom row: asymmetric loops corresponding to preserved chirality (the vortex nucleates on opposite sides in the two loop branches). Red lines: first (descending) loop branch. Blue lines: second (ascending) loop branch.

The actual local hysteresis loops measured by this technique can display different features depending on what are the chirality states of the vortices that nucleate along the first and second branches of the hysteresis loop. The four possible configurations are detailed in the right hand side of Fig. 3, where the magnetisation evolution as a function of the applied magnetic field is calculated for a square having the same characteristics as the experimental ones^21^.

In Fig. 3, representative simulated MFM images are reported for the main magnetisation states^35^ experienced by the square during a hysteresis loop, namely: positive saturation, development of the so-called C-state, vortex nucleation, vortex expulsion, C-state, negative saturation, and then back to positive saturation again. The simulated MFM images (that have been calculated for a tip-sample distance of 50 nm, in agreement with the exploited experimental conditions) are placed (not in scale) on a time axis along which the applied magnetic field is represented (in red for the first loop branch and in blue for the second one). Two magnetisation histories are illustrated: in the one in the top half of Fig. 3 the vortex nucleates at the same side of the square (the bottom edge, in this example) during both the first and the second loop branch; in this condition, the vortex chirality is inverted between the two loop branches, as can be clearly seen by comparing the two simulated MFM images corresponding to the vortex states: as for the experimental MFM images, a convenient way to compare chirality states consists in choosing an arbitrary reference point (e.g. the top-left corner of the square) and following the colour sequence (e.g. bright-dark or dark-bright). Conversely, in the history at the bottom half of Fig. 3, the vortex nucleates at two opposite sides (first bottom edge, then top edge, in this example) in the two loop branches, therefore preserving its chirality. In order to extract local hysteresis loops from these magnetic histories, one should imagine an MFM tip repeatedly scanning a selected profile of the simulated square, for all the applied field values. For each magnetisation reversal history (preserved or inverted vortex chirality), two possible choices are shown in Fig. 3, represented by the green dashed lines: the MFM tip is supposed to scan a profile located at 120 nm from either the bottom or top edge of the square. In this way, for all the magnetic fields, the selected magnetic profile of the simulated MFM image can be extracted to compose a resulting image similar to those that are experimentally obtained (see Fig. 2). These images, shown in the right part of Fig. 3, can be treated as the experimental ones, giving rise to the four representative local hysteresis loops depicted in the figure. Loops of type I and II are symmetric, because symmetric is the field evolution of the magnetisation with respect to the position of the scanning MFM tip. For type I loops the tip is imagined to scan the square at the opposite side with respect to the edge where the vortex nucleates, for each of the two loop branches. Similarly, for type II loops the tip scans the sample on the same side as where the vortex nucleates, for each of the two loop branches. Conversely, for type III and type IV loops the edge of the square at which the vortex nucleates changes from one loop branch to the other, while the tip always scans the same profile for the whole field history. For this reason, type III and type IV loops are asymmetric.

It is important to note that in hysteresis loops measured with MOKE or other magnetometric techniques the shape of the loop is always symmetric, because only the projection of the magnetisation vector along the direction of the applied magnetic field is taken into account, therefore symmetrising the result. Conversely, with local hysteresis loops measured with the technique discussed here the symmetry is broken by the choice of the scanned profile. If the profile is chosen in one of the two halves of the examined square, the symmetry is automatically broken and one of the four possible hysteresis loops would result. It is also important to point out that the qualitative appearance of the four hysteresis loops remains the same for scanned profiles that lie approximately in the first third of the square from the bottom or top edge; therefore, the choice of the scanned profile is not particularly critical when performing the experiments.

**Figure 4:**
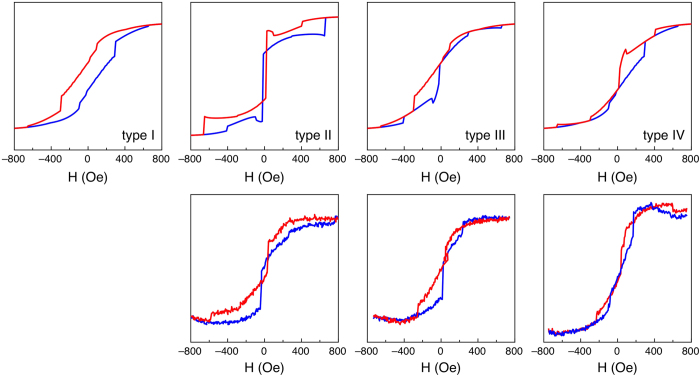
Simulated (top row) and experimental (bottom row) local hysteresis loops. Red lines: first (descending) loop branch. Blue lines: second (ascending) loop branch. The vertical axes report MFM “phase contrast” in arbitrary units, as the phase scale of the microscope does not require to be calibrated.

A selection of square dots of the array have therefore been repeatedly investigated by choosing profiles located close to either the top or bottom edge. A summary of the experimental results is reported in Fig. 4 together with a direct comparison with the calculations. The agreement between measured and simulated local loops is evident, for types II, III and IV. Interestingly, type I loops have never been experimentally observed for our sample, and type II loops turn out to be obtained most of the time, whereas an equiprobable occurrence of the four configurations would have been expected.

To understand the reason of this peculiarity, we investigate the possibility that the tip-sample interaction, which has been considered negligible so far in the simulations, actually plays a role. In fact, in Fig. 4 type I loops are missing, which correspond to nucleation of the vortex at the opposite side of the scanning tip for both loop branches, whereas type II loops, which are most commonly obtained, correspond to the nucleation of the vortex at the same side of the scanning tip for both loop branches.

**Figure 5:**
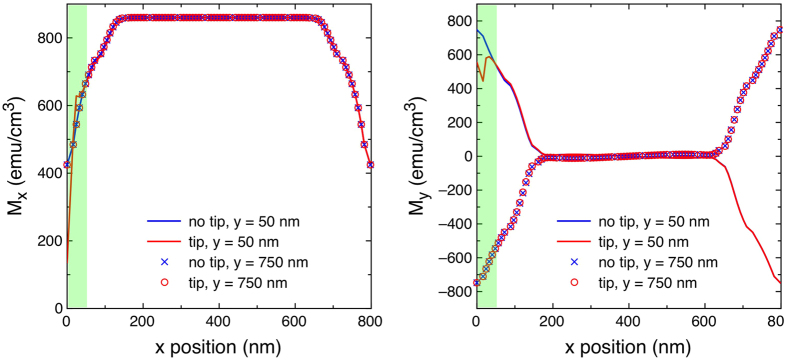
Simulated *x* and *y* components of the magnetisation of a square along two horizontal profiles, at 50 nm (lines, where the MFM tip performs the scan) and 750 nm (symbols) from the bottom edge. The magnetic field generated by the tip is either neglected (blue colour) or taken into account (red colour) in the simulations. The green shaded area shows the region that is affected by the tip-sample interaction when the tip is close to the left margin of the square (beginning of the line scan and end of the respective retrace).

Therefore, tip-sample interaction has been micromagnetically simulated by adding the magnetic stray field produced by the tip to the effective field in the Landau-Lifshitz-Gilbert equation. The tip is represented as a magnetic dipole, with its centre located in correspondence of the lift height. Its magnetic moment amplitude is assumed equal to 5.6 · 10^−18^ Am^2^ (one tenth of the nominal one for a MESP-HR tip used in experiments), in agreement with the analysis reported in ref. 41, where the stray field produced by tips of different types was estimated at a retrace distance of 50 nm. The stray field from the tip does not significantly affect the shape of the “normal” hysteresis loop, i.e. the projection of the magnetisation vector along the direction of the field. However, a close look at the *x* (parallel to the scanning direction) and *y* (orthogonal to it) components of magnetisation reveals the role played by the tip-sample interaction in driving vortex chirality. In Fig. 5 the *M*_*x*_ and *M*_*y*_ components of the simulated magnetisation are reported as a function of the tip position along *x* (*x* = 0 nm and *x =* 800 nm representing the left and right edges of the square, respectively) for two profiles respectively at 50 nm from the bottom (*y *= 50 nm) and from the top (*y* = 750 nm) edge of the square. The tip scans the square along the profile located 50 nm from the bottom edge (*y *= 50 nm), similar to the experimental conditions. Figure 5 reports the data for both zero and non-zero tip-sample interaction, when the tip is located close to the left edge of the square (*x* = 10 nm, *y* = 50 nm) and at a vertical distance from the square surface of 50 nm, as in the last configuration of pass 2 in the MFM measurements; the chosen equilibrium point corresponds to the symmetric state coming from positive saturation that immediately precedes the development of the C-state. According to the simulations, the magnetisation evolves from saturation keeping a symmetry with respect to the square axis until, at a critical field, an irreversible process takes place giving rise to the asymmetric C-state. This state, which can have either clockwise or anticlockwise chirality, univocally defines the chirality of the vortex that will nucleate at a lower field. Therefore, this specific equilibrium point, where the magnetisation is still symmetric and is just about to irreversibly jump to an asymmetric configuration, is the most useful to investigate possible effects of the tip-sample interaction in the determination of the vortex chirality.

When the tip-sample interaction is zero, *M*_*x*_ is exactly the same whether it is evaluated at *y* = 50 or *y* = 750 nm (blue line and symbols respectively). Similarly, for the same condition, *M*_*y*_ is symmetric, as can be easily verified by considering how the magnetisation is oriented in the simulated MFM images representing C-states in Fig. 3. However, when the tip-sample interaction is switched on, the corresponding *M*_*x*_ and *M*_*y*_ profiles (red line and symbols) show a weak lack of superposition, therefore meaning a breaking of symmetry, in a very small region close to the tip location, and evidenced by the shaded area in Fig. 5. These weak perturbations in the *M*_*x*_ and *M*_*y*_ components of the magnetisation are practically undetectable in the “normal” hysteresis loop, where the magnetisation vector is projected along the direction of the applied field, but are responsible for the formation of the specific C-state that will eventually cause the nucleation of the vortex on the same side of the tip. If the tip position is changed in the simulations from *y* = 50 to *y* = 750 nm, then the vortex will nucleate on the opposite side as well.

It has to be remarked that the simulations deal with an ideal system, therefore the tip-sample interaction, although weak, is able to deterministically influence the side of nucleation of the vortex and therefore its chirality. In the real sample, however, several factors can affect the breaking of symmetry, including thermal effects, imperfections in the shape, misalignment of the magnetic field. When the square dot is left free to evolve toward its magnetic remanence from saturation, with no interaction with the magnetic tip, the resulting vortex chirality is effectively random, as discussed with regard to Fig. 1. However, when the tip scans the same profile of the square while the magnetic field is applied, its interaction with the square magnetisation favours the nucleation of the vortex at the same side of the scanned profile, as discussed with reference to Fig. 4, in agreement with simulations. In fact, type II loops turn out to be the most frequently obtained, type III and IV are less frequent, and in our case type I loops have never been observed. A certain degree of randomness is still present in the real case, with the nucleation of the vortex on the opposite side of the tip, therefore leading to the appearance of type III and IV loops in a limited number of cases. Type I loops are by far the most unlikely to occur, as the vortex must nucleate on the opposite edge with respect to the tip for both loop branches, and have not been observed in our experimental results.

**Figure 6:**
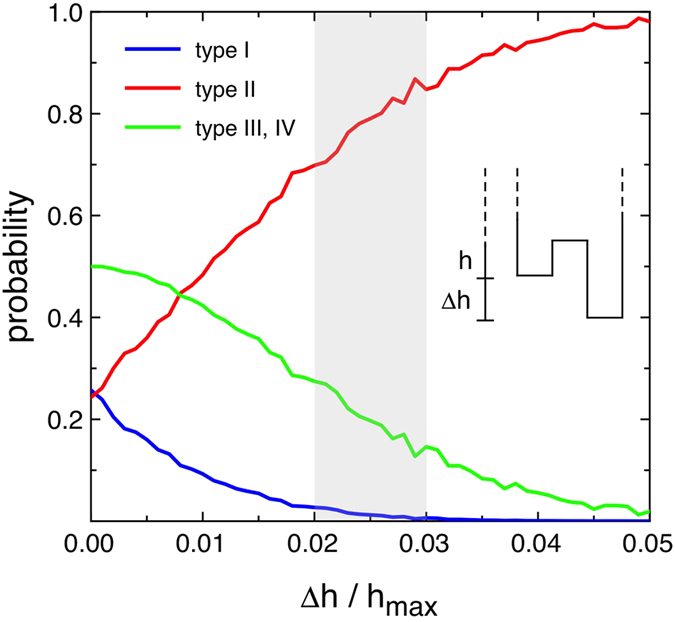
Probability of obtaining type I, II, III and IV local hysteresis loops as a function of the asymmetry between the two wells corresponding to the two different chirality states. The experimentally observed probabilities lie in the shaded area.

The results of the Monte Carlo simulations have then be used to assess whether the tip-sample interaction could be responsible for the experimentally observed probabilities of the four loop types. The results are shown in Fig. 6. For 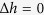, i.e. for the case with no tip-sample interaction, the system will eventually be in any of the two possible states with equal probability: therefore, each loop type will be observed in 25% of the cases. However, type III and IV loops are equivalent, as the vortex nucleates on both square sides during the loop, and their combined probability is therefore 50%. As soon as Δ*h* increases from zero, the probability of finding the system in the well that is less deep (i.e. in the state that will lead to the nucleation of the vortex on the opposite side of the tip) decreases rapidly. Type I loops, where the vortex nucleates on the opposite side of the tip for both branches, tend to become rare. Type III and IV loops, where the nucleation of the vortex on the opposite side of the tip occurs only for one loop branch, become less probable, while type II loops, where the vortex nucleates on the same side of the tip for both loop branches, as predicted by the miromagnetic simulations, become the common case. As shown in Fig. 6, a Δ*h*/*h*_max_ ratio in the interval marked by the shaded area, corresponding to a weak tip-sample interaction, is sufficient to make type I loops very improbable events, and roughly accounts for the experimental frequencies of observations of the local hysteresis loops.

It is therefore interesting to discuss why chirality states at the magnetic remanence are supposed to be equiprobable (as discussed with reference to Fig. 1), while local hysteresis loop measurements clearly indicate a symmetry breaking that favours one chirality state over the other. Indeed, when a single square, or a whole square dots array, is imaged by MFM in a remanence state, the measurement procedure consists in applying a saturating field and then removing it while the tip is scanning at a certain distance from the square (or from the closest square if the whole array is imaged), whereas the actual acquisition of the image, with the tip in close vicinity to the square(s), is performed once the vortices have already nucleated. Therefore, the influence of the tip on the square dot magnetisation is negligible. Conversely, during a local field loop measurement, the tip is already scanning the chosen profile when the magnetic field is applied and then the square is gradually brought to remanence passing through the development of the C-state. During this process, the tip will be close to the edge of the square (as in Fig. 5) twice for all applied field values (once in pass 1 and once in pass 2), inducing the weak symmetry breaking that is responsible for the nucleation of a vortex with a preferred chirality state over the other. Controlling the tip-sample interaction may be a means of affecting the probability of nucleating vortices on a given side of the square, according to Fig. 6, whereas it is more difficult to imagine an experimental procedure that would keep the MFM tip sufficiently far away from the square edges during each field step leading to a local hysteresis loop measurement not affected by the tip magnetisation, and therefore restoring the equiprobability of the four loop types of Fig. 4.

## Conclusions

Local hysteresis loops measurements, based on field-dependent MFM, on patterned magnetic square dots convey information on the chirality state of the magnetic vortex that nucleates during the magnetisation reversal process. This results in symmetric or asymmetric loop shapes depending on the respective inverted or preserved chirality of the vortex in the two loop branches. Four different local hysteresis loops are possible and equally probable, as shown by simulations, but tip-sample interactions can be exploited as a means to directly affect the vortex chirality by influencing the probability to nucleate a vortex on a specific edge of the square.

## Additional Information

**How to cite this article**: Coïsson, M. *et al*. Magnetic vortex chirality determination via local hysteresis loops measurements with magnetic force microscopy. *Sci. Rep.* **6**, 29904; doi: 10.1038/srep29904 (2016).

## Supplementary Material

Supplementary Information
