# Supplementary material for: Magnetic vortex chirality determination via local hysteresis loops measurements with magnetic force microscopy
Source: Sci Rep. 2016 Jul 18;6:29904. doi: 10.1038/srep29904 (PMC4947910; doi:10.1038/srep29904)
Supplement: Supplementary Information [file srep29904-s1.pdf]

Magnetic vortex chirality determination via local hysteresis loops measurements with magnetic force microscopy

M. Coïsson, G. Barrera F. Celegato, A. Manzin, F. Vinai, P. Tiberto

Supplementary Information

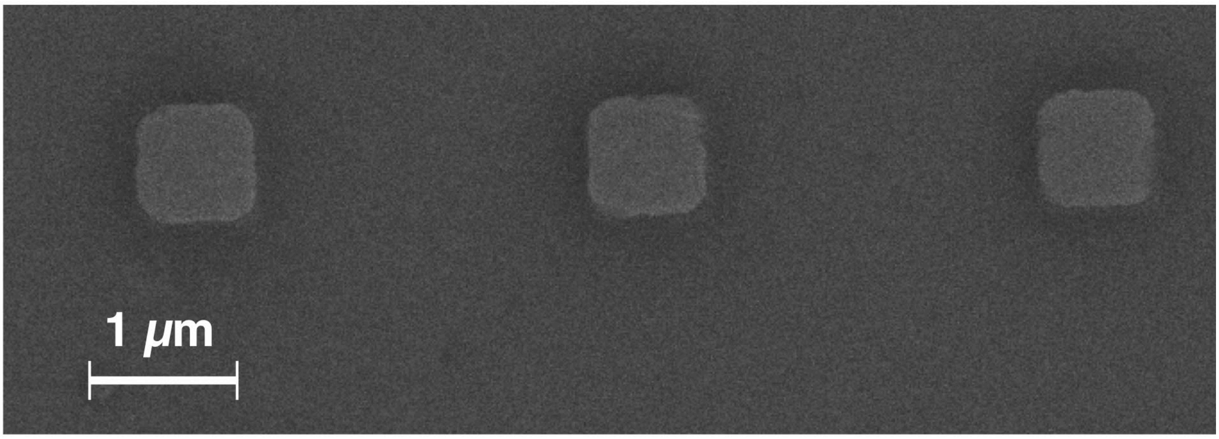

Figure S1. SEM image of a portion of the $Ni_{80}Fe_{20}$ dot array.
